# Breast Cancer Liver Metastasis: Primary tumor predictors and histopathologic characteristics of metastatic lesions

**DOI:** 10.21203/rs.3.rs-9903557/v1

**Published:** 2026-08-06

**Authors:** Pepper Schedin, Hatun Duran Cete, Jackie Phipps, Alexandra Bartlett, Michelle Ozaki, Skye Mayo

**Affiliations:** Oregon Health and Science University; Oregon Health & Science University; Oregon Health & Science University; Oregon Health & Science University; Oregon Health & Science University

**Keywords:** Young onset breast cancer, postpartum breast cancer, breast cancer liver metastasis, liver tropism, estrogen receptor conversion, replacement growth, sinusoidal endothelial cells

## Abstract

Young breast cancer patients have increased risk for liver metastasis (BCLM), implicating age-dependent organotropism. To explore potential risk factors and mechanisms of BCLM, we unbiasedly recruited 47 patients diagnosed with BCLM at a tertiary medical center, collected clinicopathologic data of the primary breast cancers, and obtained paired BCLM lesion tissue. Compared to the overall breast cancer patient population, our BCLM cohort was enriched ~4 fold for young patients (YOBC) and ~5 fold for patients diagnosed within 10 years of recent childbirth. The majority of primary breast cancer diagnoses were luminal A, low-grade and early-stage disease, clinical attributes classically associated with good prognosis. Most BCLM lesions were also ER-positive, and the dominant breast cancer growth pattern was replacement. Immunohistology staining infers utilization of existing liver sinusoids, consistent with a passive mechanism of metastatic invasion. In sum, these data suggest that the liver readily supports metastatic establishment of early-stage, luminal A breast cancer cells. These data also support a growing body of literature linking postpartum liver biology to increased risk for BCLM, providing potential insight into the observed age-dependent organotropism of BCLM. Improved understanding of reproductive risk factors for BCLM may improve patient risk stratification and outcomes overall.

## Background

Mortality from breast cancer is largely due to metastatic spread, leading to distant organ failure and death^1^. The impact of metastatic disease is evident from 5-year overall survival which is > 99% for patients diagnosed with localized invasive ductal carcinoma, but only 32% for patients diagnosed with metastatic disease^2^. Although recent advancements in early detection and treatment have decreased breast cancer deaths overall^2^, survival improvements for patients with young onset breast cancer (YOBC) are lagging^3^, with recent data highlighting the propensity for metastasis in YOBC^4–6^. Further, when defined as a diagnosis before 50 years of age, YOBC accounts for 16% of all US cases^7^, and is the leading cause of death in U.S. females in this age group^8^. A 2024 study of the National Surveillance, Epidemiology, and End Results (SEER) registries found increasing rates of YOBC across all races and ethnicities^5,8^. This rising incidence coupled with poor prognosis in the YOBC population calls for an improved understanding of drivers of metastasis in young patients.

When compared to postmenopausal cases, young women have higher incidence of breast cancer with poor prognostic features including estrogen receptor negative (ER-negative) tumors, high proliferation rates, and later stage at presentation^9–12^. These poor prognostic tumor features, combined with potential delayed diagnosis due to many patients being too young for population-screening, are considered important drivers of breast cancer mortality in YOBC^9–11,13^. However, more recent studies find that young women diagnosed with early-stage ER-positive breast cancers also have high rates of metastatic progression^14–16^. Notably, more YOBC patients die from ER-positive than ER-negative disease^17^. Also, when the potential for delayed diagnosis is controlled, by normalizing tumor size and stage across age groups, poor prognosis of YOBC persists^3^. Combined, these data suggest that poor prognostic tumor subtypes and delayed diagnosis may not account for the majority of YOBC metastasis, as previously thought

More recently, postpartum host biology has been strongly implicated in breast cancer metastasis in young women. A meta-analysis of 41 studies found that young women diagnosed postpartum were at highest risk of disease progression independent of tumor subtype [HR 1.86; 95% CI 1.17–2.93]^18^. Another study reported that young, nulliparous patients did not have poorer prognosis when compared to older women, whereas young postpartum breast cancer (PPBC) patients had poorest outcomes compared to both older women and young, nulliparous women^19^. Other studies report that breast cancers diagnosed up to 10 years post-childbirth specifically associate with a 3- to 8-fold increased risk of liver metastasis, with increases in bone, lung or brain metastases not observed^17,20,21^. Thus, a growing body of evidence suggests that biologic changes associated with recent childbirth may influence the spread of breast cancer to the liver.

Herein, we evaluated patient demographics and tumor clinicopathologic characteristics from a patient population diagnosed with BCLM at single academic medical center. We find our findings align with reported data, including enrichment of YOBC, PPBC and ER-positive cases. Further, our results are consistent with the liver being a highly permissive environment for BCLM, as the majority of primary breast cancers that progressed to liver metastasis were luminal A and early-stage, and growth within the liver was replacement, inferring utilization of existing liver vasculature rather than gain of tumor cell intrinsic angiogenic capacity. Finally, our observation that BCLM patients were frequently young and postpartum provides rationale for increased research into whether recent childbirth increases risk for breast cancer metastasis to the liver.

## Methods

This was a single institution, cohort study to investigate the clinicopathological and tumor characteristics of breast cancer patients with liver metastasis. The study included 47 cases with available BCLM tissue, comprised of retrospective cases obtained from OHSU’s Biolibrary (n = 20), and prospective cases (n = 27) (**Supplementary Fig. 1**). Biolibrary cases of BCLM were diagnosed between 1995 and 2013. Prospective cases were comprised of patients with first diagnosis of liver metastasis (n = 12) and patients with BCLM progression (n = 15), recruited at time of surgical scheduling between 2017 and 2024. All clinicopathologic data were obtained through chart review of patient electronic medical records, with IRB approval. This cohort study is reported in compliance with the Strengthening the Reporting of Observational Studies in Epidemiology (STROBE) guidelines.

### Study Oversight

This study was approved by the Oregon Health & Science University (OHSU) Institutional Review Board (IRB). Retrospective cases (n = 20) obtained through the OHSU BioLibrary were from patients who consented to use of their tissues for future research. Prospective biospecimens and data were acquired and analyzed under the OHSU IRB-approved protocols Molecular Mechanisms of Tumor Evolution and Resistance to Therapy (MMTERT, IRB#16113)^22^ (n = 15) and Exploration of the Liver Metastatic Niche in Breast Cancer Patients with Metastases to the Liver (IRB#19164) (n = 12). Participant eligibility was determined by the enrolling physician and/or Oregon Clinical and Translational Research Institute (OCTRI) clinical coordinator and informed written consent was obtained from all subjects. The study adhered to the ethical principles of the Declaration of Helsinki and followed the International Council on Harmonization guidelines on Good Clinical Practice, in compliance with all applicable laws, regulatory requirements, and conditions mandated by regulatory authorities and/or institutional review boards.

### Inclusion/Exclusion criteria

Patients were included if they were at least 18 years of age, had a diagnosis of primary breast cancer (stage I-III) that progressed to liver metastasis, or were stage IV patients with liver metastasis at time of primary breast cancer diagnosis. Exclusion criteria were history of malignancy other than breast cancer; presence of liver disease other than breast cancer metastasis to the liver, known autoimmune conditions, chronic systemic steroid use, use of immunomodulatory prescription drugs for any medical condition, presence of other comorbidity conditions known to impact immune function, and for prospective patients only, known bleeding diathesis.

### Variables and Data Sources

The following clinical and demographic information were abstracted from the electronic medical records (EMR): age at the time of primary breast cancer diagnosis delineated into three groups (≤ 45years, 45–60 years, > 60years); menopausal status determined according to the reported median age of natural menopause in United States (pre-menopausal ≤ 51y and post-menopausal > 51y)^23^; parity status defined as at least one full term childbirth (parous group) or no full term childbirth (nulliparous group); time between breast cancer diagnosis and last childbirth, with cases delineated as PPBC when diagnosed with primary breast cancer within 10 years of last childbirth and non-PPBC (diagnosed after 10 years of childbirth or nulliparous), as previously defined^17^. PPBC sub-analyses were restricted to premenopausal cases to ensure that the cases in PPBC and non-PPBC were in similar age ranges. We estimated the US population parity status by finding the percentage of woman who have children at 15–49 years of age^24,25^, and weighted each group’s parental rate by its share of the total female population, as previously described^26^. Primary breast cancer clinical characteristics included hormone receptor status (estrogen receptor (ER), progesterone receptor (PR)), and human epidermal growth factor 2 status (HER2); pathological tumor grade (1, 2, 3); and stage (I, II, III and IV) designated according to the tumor node metastasis (TNM) classification system of American Joint Committee on Cancer (7th edition, 2010). Tumor biological subtypes were delineated as luminal A (ER + PR+HER2-), luminal B (ER + PR-HER2-), luminal-HER2 (ER + PR±HER2+), HER2 (ER-PR- HER2+) and triple negative breast cancers (TNBC) (ER-PR-HER2-).

For all BCLM tumors, hormone receptor status data were collected through electronic medical records and for the cases with missing ER status we stained and assessed for ER status as previously described^27^. BCLM differentiation grade and histopathologic growth pattern were assessed by hematoxylin and eosin stained FFPE BCLM tumors. We adapted the Nottingham Histologic Score System^28^ to describe the differentiation grade of the BCLM tumors that included tubular and glandular differentiation, nuclear pleomorphism (size and shape of nuclei), and mitotic count to delineate differentiation grade into well, moderately or poorly categories.

Histopathologic growth patterns of metastatic lesions were determined by assessing the adjacent normal liver-tumor interface, based on established scoring for liver metastasis in colorectal cancer^29–31^. Growth patterns were delineated into replacement, where tumor cells are arranged in continuity with the hepatocyte cords and or in continuity with the hepatic sinusoids; pushing, where the tumor presses against the normal liver causing liver compression; and desmoplastic, where a stromal rich rim separates the tumor mass from the adjacent normal liver. Tissues from cases that did not contain normal adjacent liver- tumor interface were excluded from the metastatic growth pattern analysis. The differentiation state and histologic growth pattern of BCLM assessments were determined independently by two researchers blinded to patient data, and cases with disparate results were evaluated by a third independent reviewer, with consensus data reported. The Ki67 index for BCLM lesions was collected through the electronic records of patients’ pathology immunohistochemistry (IHC) reports. For BCLM cases with missing Ki67 data (n = 18), we stained FFPE thin sections and assessed percentage of Ki67 positive cells to total number of tumor cells based on established criteria^32^. For tumors with Ki67 heterogeneity, the highest percentage of Ki67 was reported. Gata binding protein 3 (GATA3), hepatocyte paraffin 1(Hep Par 1), collagen IV (Col IV) and fibronectin (FN) protein were assessed by multiplex IHC (mIHC) staining and Col IV, FN and Endoglin/CD105 protein assessed by immunofluorescence (IF) as described below.

### Multiplex Immunohistochemistry Methods

Multiplex IHC staining was performed as previously described with modifications^33^. Thin sectioned FFPE tissues adhered to slides (Tanner Scientific Microscope Slides, Catalog no: TNR WHT45AD, Mercedes Scientific) were deparaffinized, rehydrated, hematoxylin stained, and images captured prior to antibody cycles. Slides were deparaffinized in a 60°C oven for 1 hour, then soaked in xylene four times for five minutes each time. Slides were rehydrated by dipping 20 times in decreasing percentages of EtOH (100% x2, 95% x2, 70% x2, 50% x2, diH_2_O x2). A heat mediated antigen retrieval was performed with ethylenediaminetetraacetic acid (EDTA) Decloaker pH 9.0 (Biocare Medical, 5082398) in a pressure cooker for 5 minutes at 125°C. Slides were allowed to cool at room temperature for 15 minutes, followed by 5-minute washes (distilled water (diH_2_O) x2, Tris-Buffered Saline with Tween-20 (TBST) x1). An endogenous block was applied to the tissue for 20 minutes (3% H_2_O_2_ in MeOH), followed by TBST washes (2 minutes x3). Slides were stained for hematoxylin (Dako, S3301) for 2 minutes and allowed to blue in TBST for 2 minutes. Slides were temporarily cover slipped using TBST and No. 1 thickness glass cover slip (Epredia, 12460S) and scanned. After scanning, temporary coverslips were removed by soaking in TBST, with shaking, for 15 minutes. Hematoxylin was removed using 1% SDS (Sodium Dodecyl Sulfate) Glycine Elution buffer wash at room temperature for 5 minutes. Antibodies were diluted (Dako, S3022) and all primary antibodies were incubated for 60 minutes and washed in TBST (2 minutes, x3), and all secondary antibodies were incubated for 30 minutes and washed in TBST (2 minutes x3) at room temperature. 3-amino-9-ethylcarbazole (AEC) was used as a chromogen (Vector Laboratories, SK4200) and incubated for 20 minutes and rinse with diH_2_O. Hematoxylin was applied to the tissue as a counterstain for 1 minute. After each antibody, stained tissues were scanned using the Aperio AT2 slide scanner (Leica Biosystems). Following scanning, AEC chromogen was removed with 1×70% and 1×100% alcohol wash for 2 minutes each, with 1–2 primary antibodies per cycle (**Supplementary Table 1**). Between cycles, primary and secondary antibodies were removed using 20% SDS-glycine pH 2 at 50° 120 minutes for Hep Par 1 and Col IV, 60 minutes for Ki67 and 30 minutes for GATA3 and ER. Secondary-only and isotype controls were used to confirm antibody striping after each cycle. Each case slide also included a human tissue microarray (TMA) with positive and negative control tissue to confirm primary antibody specificity on a case-by-case basis. The TMA included normal human liver, primary breast cancer, and adjacent normal breast tissue. After serial staining and image capture, image alignment and extraction were performed using the SURF algorithm in the Computer Vision Toolbox of Matlab version R2018b (The MathWorks, Inc, Natick, MA, USA). Color deconvolution was performed in Fiji and pseudo-colored multiplex images were created in Aperio ImageScope v12.1.0.5029. For quantification of IHC signal, tumor borders were annotated by a clinician blinded to case data, and analyses performed on tumor only. Signal quantifications of stains were performed using modified color deconvolution algorithms in Aperio ImageScope v12.1.0.5029 as described^33^. Data are presented as percent positive nuclei (ER, GATA3 and Ki67) and percent positive areas (Hep Par 1, Col IV, FN).

### Immunofluorescence Staining

Thin sectioned FFPE tissues adhered to slides (Tanner Scientific Microscope Slides, Catalog no: TNR WHT45AD, Mercedes Scientific) were deparaffinized and rehydrated using xylene and ethanol as described above. Pressure cooker (BioSB BSB7087) heat mediated antigen retrieval was performed with 1X Abcam Antigen Retrieval Tris-EDTA Buffer, pH 9.0 (Abcam ab93684) at high pressure and 125°C for 20 minutes. Slides were allowed to cool in buffer on the benchtop for 20 minutes. Slides were then blocked with Background Sniper Protein Block (Biocare Medical BS966) for 30 minutes at room temperature. Antibodies were diluted in Dako Antibody Diluent (Dako S302283–2) (**Supplementary Table 1)** and incubated overnight at 4C. After antibody staining, slides were incubated with TrueView Autofluorescence kit (Vector Labs, SP-8400–15) for 1 minute, incubated with 5ug/mL DAPI in PBS Sigma-Aldrich D9542–5MG) for 10 minutes, mounted with VECTASHIELD Vibrance^®^ Antifade Mounting Medium (Vector Labs, H-1700–10) and cover slipped. Images were taken using a Zeiss Apotome 3 microscope.

### Collagen IV Scoring

To qualitatively assess Col IV remodeling at the tumor border, we selected 3 ER-positive, chemo naïve BCLM cases with clear tumor and adjacent normal liver interface. Two non-overlapping regions of interest (ROI) were selected from each case. Using Aperio ImageScope software, the tumor border was marked using GATA3 IHC to identify the tumor invasive edge. The length of adjacent normal-tumor interface analyzed ranged from 840 μm to 1470 μm with an average of 1215 μm across the 6 total ROIs. The tumor border annotation was then copied onto the corresponding Col IV IHC image. Next, using Aperio, we delineated ~ 100 μm in either direction of the tumor border. Within this 200 μm region, we classified the Col IV staining patterns into three descriptive categories: sharp, defined Col IV stain that closely and continuously aligned with the sinusoidal shape (Category 1), and as occurs in normal, non-tumor liver; Col IV stain aligned to the sinusoidal shape but with modest diffuse staining pattern (Category 2); and wider and more diffusely stained Col IV that did not closely align with sinusoidal shape (Category 3). Total number of data points for each ROI ranged from 15–30. We combined data from individual ROIs to calculate category distributions in adjacent normal liver and BCLM tumor.

### Sample Blinding

For all analyses, all cases were assigned a unique de-identified study ID, and the research team was blinded to patien’s name and medical record number. For all pathologic and immunohistochemical data collection and quantification, the research team was blinded to the patient demographic and clinical data including patient age, parity status, and tumor ER, stage and grade status.

### Statistical Methods

The sample size was determined by number of available cases of BCLM recruited to the study during the data collection period (2019–2024). Study variables included age at primary diagnosis, menopausal status, parous status, postpartum breast cancer status, breast cancer and liver metastasis hormone status, grade, stage and biological subtype, differentiation grade and growth pattern of liver metastatic lesions. Descriptive statistics were reported as frequencies and percentages. Group comparisons were performed using the Mann–Whitney U test, Welch’s t-test, Kruskal Wallis test and/or ANOVA-test based on study design. A p-value of less than 0.05 was considered statistically significant. Statistical analyses were conducted in SAS version 9.4 (SAS Institute Inc., Cary, NC) and data visualization was performed using GraphPad Prism version 10.4.1 (627).

## Results

### Patient demographics at the time of primary breast cancer diagnosis

Of the 47 cases available for analysis, we found 38% (18 of 47) were ≤ 45 years of age, 49% (23 of 47) were between 45–60 years of age and only 11% (5 of 47) were > 60 years of age, with 2% age unknown (1 of 47) (Table 1). These observations are consistent with an approximate 4-fold enrichment in YOBC cases in the BCLM cohort compared to national averages^34^. Of note, the overall breast cancer population ≤ 45 years of age at OHSU during the study period of 2019–2024 was 9% **(Supplementary Table 2)**, in alignment with YOBC incidence in U.S. women, reported at 11%^34^. Furthermore, 57% of all cases (27 of 47) were pre-menopausal, which is also high compared to the 24.7% pre-menopausal cases reported in the US^34,35^. Of the 43 women with known childbearing data, 91% (39 of 43) had at least one childbirth, slightly elevated compared to the 82% expected in the general population of same age range, and as described in methods^24–26^. We found 28% (13 of 47) of our cohort were PPBC, 53% (25 of 47) were non-PPBC, and 19% (9 of47) were of unknown parity status. Of the women ≤ 45 years of age, 72% (13 of 18) were PPBC, 17% (3 of 18) were non-PPBC, and 11% (2 of 47) were unknown parity status (Table 1). Thus, our cohort was enriched ~ 5 fold for patients diagnosed within 10 years of childbirth, and our young women cohort enriched ~ 2 fold compared to previously reports of patients diagnosed with PPBC^17,36,37^.

### Clinical characteristics of primary breast cancer diagnoses

**Figure 1:**
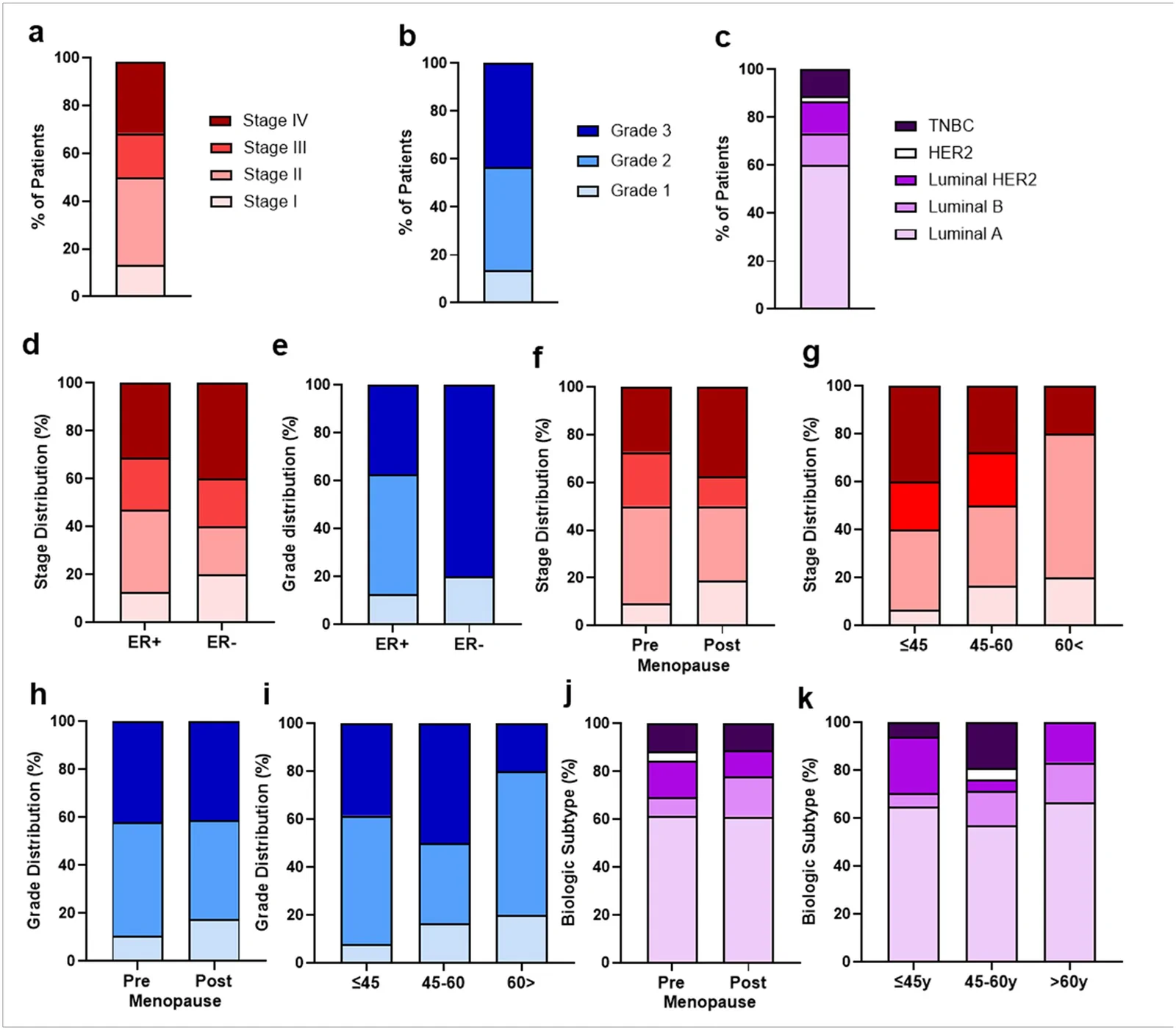
Clinical characteristics of primary breast cancers that progressed to BCLM. Distribution of primary diagnoses by **a)** tumor stage (n=38), **b)** tumor grade (n=37) and **c)** biological subtype (n=45). Distribution of tumor **d)** stage and **e)** grade delineated by primary breast cancer ER status (n=37). Tumor stage, grade and biologic subtype distribution by **f, h & j)** menopausal status (n=38–36-43 cases/group, respectively) and **g, i, & k)** patient age (n=38–36-44 cases/group, respectively). Data expressed as % of cases per group.

We next assessed whether at time of primary breast cancer diagnoses, ER-negative or HER2-positive subtypes were increased in BLCM patients, as might be predicted based on the increased risk for metastasis compared to ER-positive breast cancer subtypes^38,39^. Of the primary breast cancer diagnoses, 85% (40 of 47) of the tumors were ER-positive (Table 2). Fifty-seven percent (27 of 47) of the tumors were luminal A (ER + PR+HER2-), 13% (6 out of 47) luminal B (ER + PR-HER2-), and 13% (6 of 47) Luminal HER 2 (ER + PR± HER2+) (Table 2). Of the ER-negative cases, 11% (5 of 47) were triple negative (ER-PR-HER2-) and only 2% (1 of 47) were HER2+ (ER-PR-HER2+) (Table 2). Of cases where tumor stage was available, 50% (19 of 38) were diagnosed at early stages (I-II) (Table 2 and Fig. 1a). When excluding stage IV patients at time of diagnosis, 73% (19 of 26) of cases were diagnosed with early-stage (I-II) breast cancer (Table 2). Further, 57% (21 of 37) were diagnosed with lower grade tumors (Grades 1–2) (Table 2 and Fig. 1b); and 60% (27 of 45) were luminal A (Table 2 and Fig. 1c). Of note, for these cases, which all led to BCLM, the distribution of primary breast cancer stage (Fig. 1d) and grade (Fig. 1e) did not differ by tumor ER status, although the number of ER-negative cases is small. We next evaluated whether stage (Fig. 1f and Fig. 1g), grade (Fig. 1h and Fig. 1i) or biologic subtype (Fig. 1j and Fig. 1k) at time of breast cancer diagnosis differed by menopausal status or patient age, as may be predicted based on enrichment for later stage, higher grade, and increased estrogen receptor-negative breast cancers observed in younger women overall^40^. We did not observe differences in these prognostic tumor categories when delineated by menopausal status or patient age.

In sum, at time of primary breast cancer diagnoses, most BCLM cases had favorable prognostic breast cancer criteria, i.e., luminal A, ER-positive, lower grade and early-stage disease^39,41^. Further, we did not observe any differences in distribution of tumor stage, grade or subtype by menopausal status or patient age, even though our cohort is enriched in young women. These data may suggest that liver biology in young and premenopausal women, rather than tumor intrinsic characteristics, may be a primary determinant of liver metastasis in this cohort.

### Tumor characteristics of the breast cancer liver metastatic lesions

We next assessed if disease progression from ER-positive primary breast cancer to ER-negative BCLM was common, as ER receptor conversion between primary and paired liver metastatic lesions has been variously reported between 14% −35%^42–44^. We found that ER-positive expression was maintained in BCLM lesions in 90% of cases (36 of 40) (Table 3). However, PR conversion from PR-positive in the primary to PR-negative in the BCLM was common, occurring in 57% of ER-positive cases (13 of 23), similar to previous reports^42,45^ (Table 3). Of note, in 4 out of 10 ER + PR^−^ primary breast cancer cases, we observed gain of PR in the BCLM lesions. Of the ER-PR- primary breast cancer cases (n = 6), all retained negative ER and PR staining in the liver (Table 3). In summary, the majority of ER-positive primary breast cancers retained ER-positive status in their metastatic liver lesions, but loss of PR expression was common.

**Figure 2:**
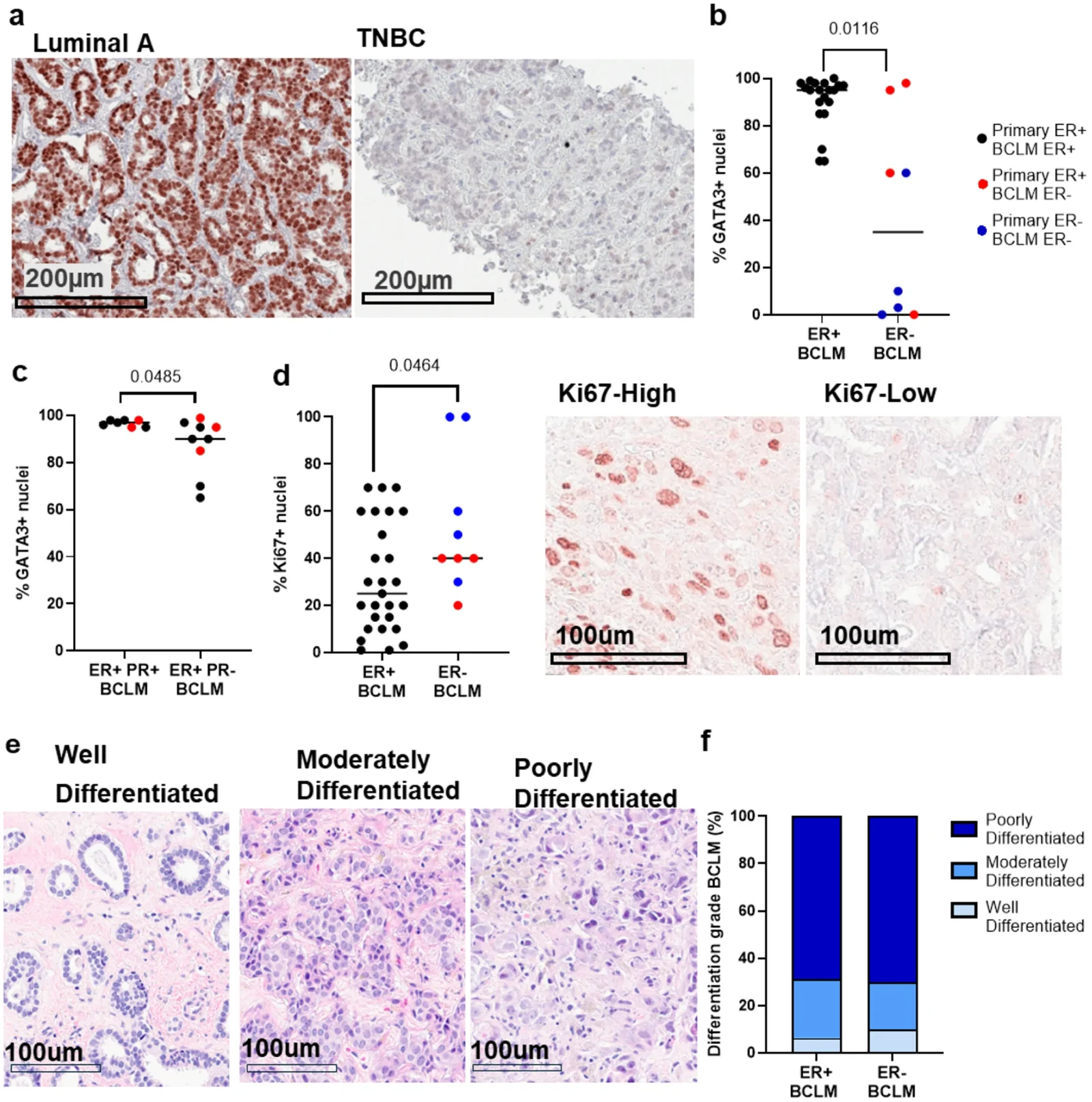
Characteristics of BCLM tumors. **a)**Immunohistochemistry (IHC) stained images of the luminal subtype biomarker GATA3, showing high expression in a representative luminal A case (left image) and low expression in a representative TNBC case (right image). Gata 3 stain is brown, scale bar is 200 μm. **b)** Quantitation of GATA3 stain delineated by ER status of the BCLM tumors, with further delineation by ER status of the primary tumor. Black dots depict ER+ primary tumors that retained ER+ in the liver metastatic site; red dots depict ER+ primary cancers that lost ER expression in the matched BCLM; blue dots depict primary ER-negative cases that remained ER-negative in the liver. ER+ (n=20) and ER- (n=8) cases, *P<0.05, Mann-Whitney, two tailed t-test. **c)** Quantitation of GATA3 staining in BCLM tumors delineated by ER+PR+ (n=7) and ER+PR- (n=9) subtypes, *P<0.05, Welch’s t (8.27) =2.31, two-tailed. **d)** Quantification of Ki67 staining by IHC in BCLM tumors delineated by ER+ (n=27) and ER- (n=9) status with representative cases for high and low Ki67 expression (brown stain), *P<0.05, Mann-Whitney, two tailed t-test. e) Representative H&E images showing differentiation state of BCLM tumors grouped by morphology into well differentiated (left), moderately differentiated (middle) and poorly differentiated (right) tumors, scale bar is 100 μm. f) Distribution of BCLM differentiation grade of BCLM tumors delineated by ER+ (n=32) and ER-(n=10) status.

Given the high abundance of ER-positive lesions in the BCLM cohort, we next evaluated for GATA3 protein expression (Fig. 2a), a transcription factor required for cell fate specification of mammary luminal cells^46^, and a biomarker for breast cancers of luminal origin^47^. All but one metastatic lesion derived from ER-positive primary breast cancers had strong expression of GATA3 in the corresponding BCLM tumor (Fig. 2b). We also found high GATA3 staining in ER-negative liver metastatic lesions derived from patients originally diagnosed with ER-positive primary breast cancer, consistent with these ER-negative BCLM lesions being luminal in origin (Fig. 2b, **red circles**). Of the 4 ER-negative primary breast cancers, GATA3 expression at the metastatic site was only observed in one case, suggesting that the three ER-negative and GATA3-negative cases were non-luminal in origin (Fig. 2b, **blue circles**). We did not find any differences in GATA3 expression in BCLM lesions when delineated by menopausal status or patient age **(Supplementary Fig. 2a-b**). Because PR expression is dependent on ER signaling, we next looked for associations between PR and GATA3 expression. In BCLM lesions, GATA3 expression was modestly lower in ER + PR- lesions compared to ER + PR+ lesions (Welch’s t (8.27) = 2.31, p=.0485, two-tailed), (Fig. 2c). These observations suggest co-reductions in the luminal biomarkers GATA3 and PR in a subset of ER-positive BCLM lesions.

High tumor cell proliferation also correlates with breast cancer progression and is elevated in primary breast cancers in young compared to older patients^12^. We measured protein expression of the proliferation biomarker Ki67 in the BCLM lesions and found Ki67 expression in ER-negative lesions was significantly higher than in ER-positive lesions (non-parametric Mann Whitney test, *U = 62, p=.046*) (Fig. 2d). However, the wide Ki67 stain variation within groups resulted in Ki67 being insufficient to delineate between ER + and ER- cases at the individual case level. We also found lower Ki67 expression in ER-negative BCLM cases derived from primary ER-positive tumors, data also consistent with the luminal origin of these BCLM lesions (Fig. 2d, **red circles)**. Of note, Ki67 levels in the BCLM cases did not associate with PR status of the primary breast cancer, menopausal status, or patient age (**Supplementary Figs. 2c-e**), respectively.

Differentiation state of the BCLM lesions (n = 42) also revealed wide variability (Fig. 2e). Sixty-nine percent of the BCLM lesions (29 of 42) were poorly differentiated, consistent with prior studies^48^. However, it is notable that a full 30% of the BCLM lesions displayed breast ductal histology consistent with highly differentiated mammary tumors (Fig. 2e). Poorly differentiated tumors were observed regardless of ER status of the metastatic lesion (Fig. 2f). Associations between highly differentiated tumor status and ER-negative primary tumors could not be assessed due to the low number of ER-negative cases overall. With respect to associations between Ki67 expression and differentiation status in liver lesions, none were found (**Supplementary Fig. 2f)**. Finally, we investigated whether BCLM lesions from PPBC patients had distinct tumor phenotypes as might be predicted if tumor intrinsic biology were driving the high prevalence of liver metastasis observed in PPBC patients. In PPBC cases, we did not observe significant enrichment in ER-negative cases, nor differences in GATA3, Ki67 or differentiation status (**Supplementary Fig. 3a-d**).

### Assessment of histologic growth pattern

**Figure 3:**
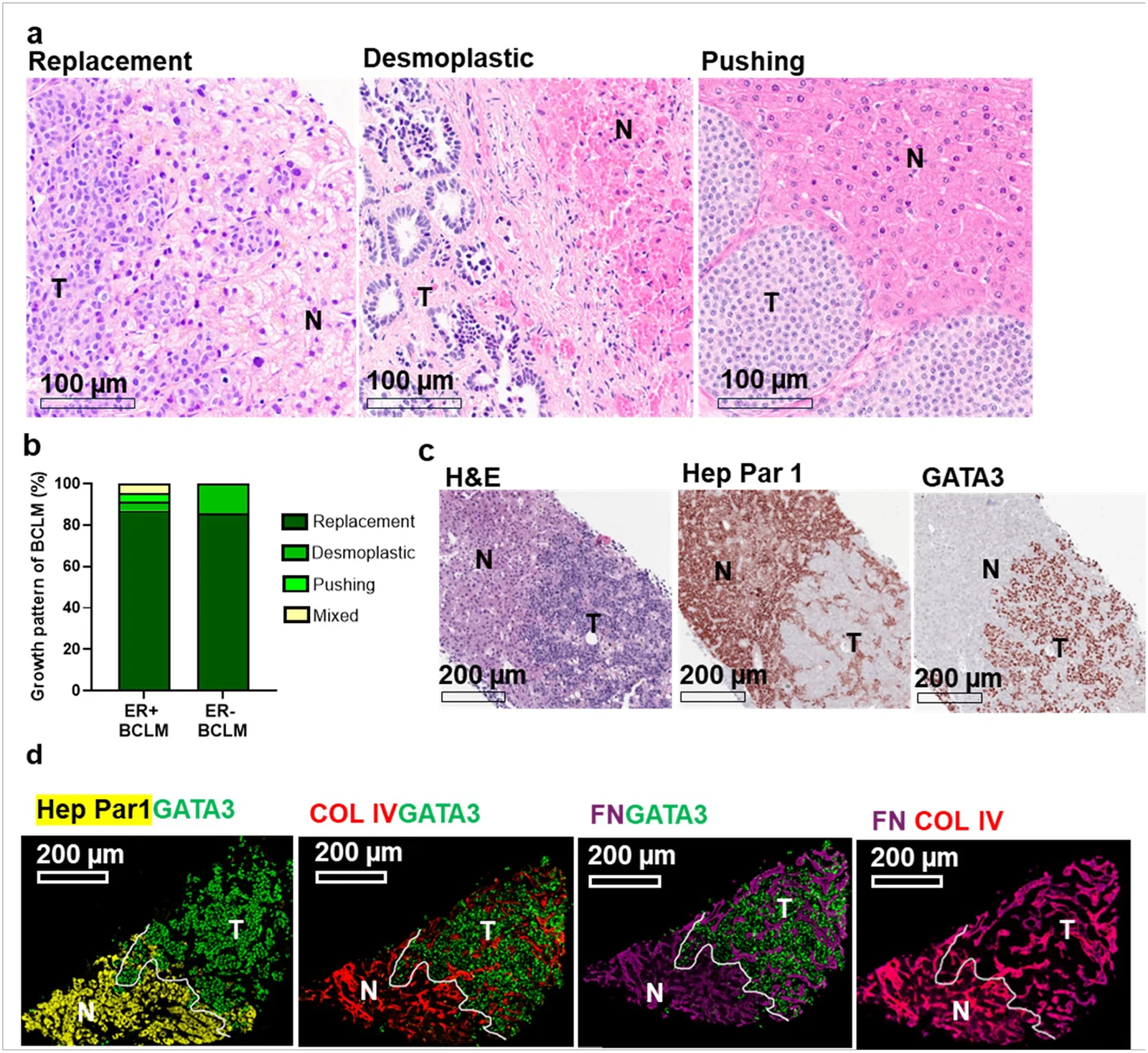
Histopathologic growth pattern of BCLM tumors. **a)**Representative H&E-stained images of three growth patterns observed in BCLM tumors: replacement (left), desmoplastic (middle), and pushing (right). Scale bar is 100 μm. T=Tumor, N=Normal adjacent liver. **b)** Percentages of replacement, desmoplastic, pushing and mixed type growth pattern according to BCLM tumor ER status (n=30). **c)** BCLM case with representative replacement growth depicting the interface between the adjacent normal liver and leading tumor edge, by H&E staining (left), mIHC staining of Hep Par1 for hepatocytes (middle) and GATA3 staining for tumor cells (right). Scale bar is 200 μm. **d)** Low magnification image of BCLM depicting the interface between the adjacent normal liver (N) and leading tumor edge (T), stained for Hep Par 1-GATA 3, Col IV-GATA3, FN -GATA3, and Col IV-FN (from left to right, respectively). Hep Par 1 (yellow), GATA 3 (green), Col IV (red), FN (purple), scale bar is 200 μm.

To obtain insight into potential mechanisms by which breast cancer cells establish liver lesions, we evaluated breast cancer growth patterns within the liver. We delineated tumor growth patterns into replacement, desmoplastic, and pushing categories as previously described^29–31^, with representative growth patterns shown (Fig. 3a). We found that the dominant histological tumor growth pattern was replacement (87%, 26 out of 30) in both ER-positive and ER-negative BCLM lesions (Fig. 3b). Because replacement growth is reported to require co-option of the existing liver vasculature^49^, we next stained representative replacement growth cases for GATA3 to delineate breast cancer cells, Hep Par 1to delineate hepatocytes (Fig. 3c), and fibronectin and collagen IV to delineate sinusoidal extracellular matrix proteins (Fig. 3d)^50^. We found that in many BCLM lesions, the FN and Col IV staining patterns were very similar between tumor and the adjacent normal liver (Fig. 3d). Morphological assessment of these leading tumor edges revealed that in 56% (9 out of 16) of replacement cases, Col IV staining was highly similar between tumor and adjacent normal liver.

**Figure 4:**
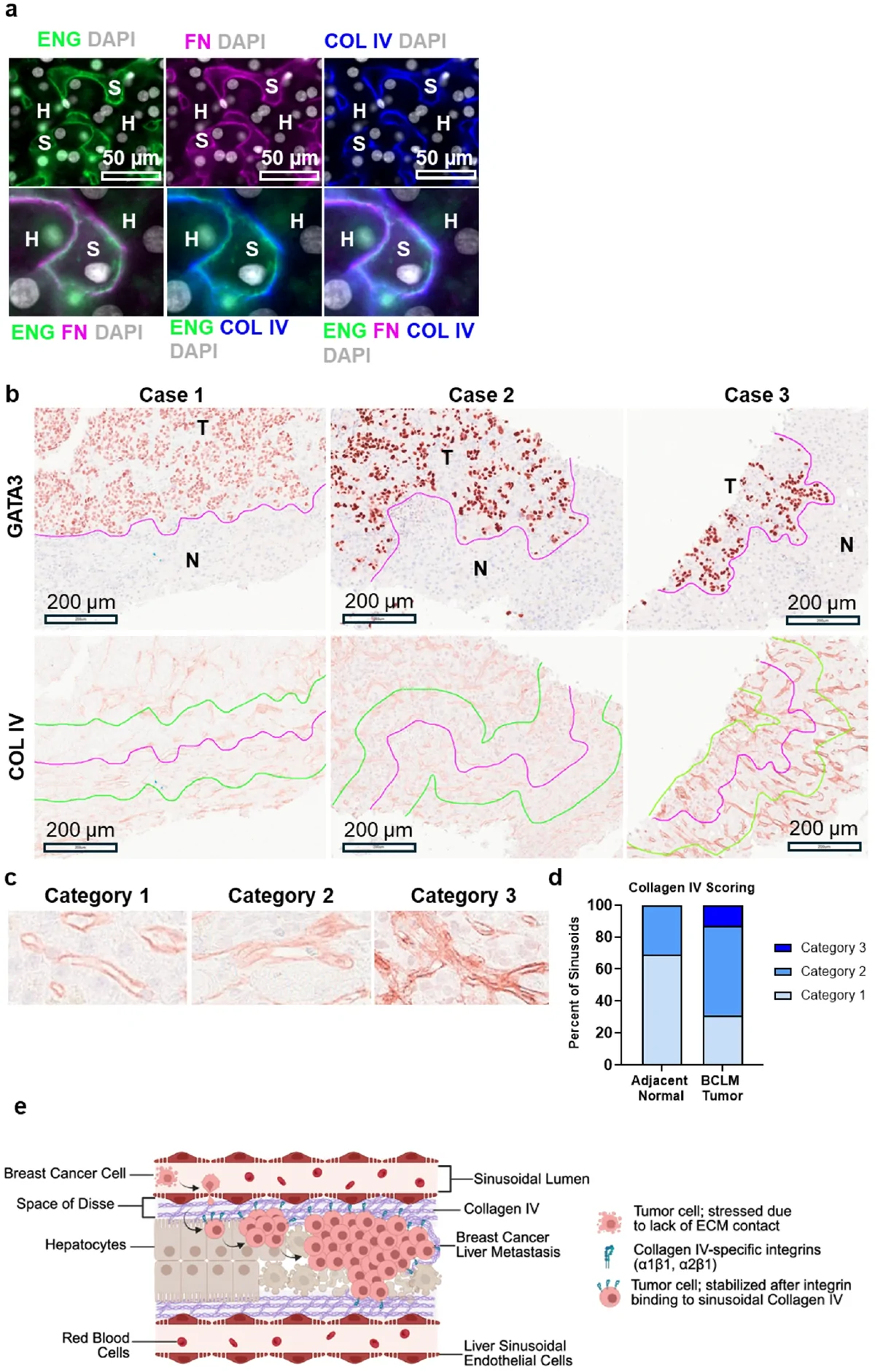
Evidence for tumor cell co-option of normal liver vasculature. Immunofluorescence (IF) staining of normal human liver sinusoids showing extracellular matrix proteins collagen IV (Col IV) and fibronectin (FN) closely and specifically co-localize with sinusoidal vasculature. **a) upper panel**, DAPI stained (grey) images of sinusoidal endothelial cells with single channel marker endoglin (ENG) (green, left image), FN (magenta, middle image), Col IV (blue, right image). S marks sinusoid lumen and H marks hepatocyte cords, scale bar is 50 μm. **Lower panel**, magnified and merged channel images of ENG plus FN (left image), ENG plus Col IV (middle image), and ENG plus FN and Col IV (right image), scale bar is 200 μm. Strategy for assessing sinusoidal associated Col IV in normal liver versus tumor border. **b) upper panel**, mIHC images depicting GATA3 staining from 3 representative ER+ BCLM tumors with replacement growth pattern. GATA3 staining was used to determine the border between tumor (T) and adjacent normal liver (N), depicted by magenta line. **Lower panel**, mIHC staining for Col IV for each case showing the tumor border in magenta, as well as green lines demarcating a 100 μm margin on either side, scale bar is 200 μm. **c)** Representative images each of the three categories of Col IV IHC staining patterns used to score Col IV structure at tumor-adjacent normal liver interface: Category 1 (normal liver), Category 2 (very modest Col IV diffusion), and Category 3 (modest Col IV diffusion). **d)**Quantification of Col IV IHC staining patterns observed within 100μm of tumor and adjacent normal liver (n=3 ER+ BCLM tumors).

Because immunofluorescence (IF) has higher spatial resolution than IHC, we next utilized IF to further evaluate the spatial relationships between the liver sinusoidal endothelial cells and fibronectin (FN) and collagen IV (Col IV). Within normal human liver, sinusoidal endothelial cells stained robustly for endoglin, a biomarker of liver sinusoidal endothelial cells^51^, (Fig. 4a, upper left panel).We also observed that FN and Col IV are closely and specifically associated with the basal side of the sinusoidal endothelium, within the space of Disse (Fig. 4a, middle and right panels). We predicted that modest ECM remodeling would occur at the tumor leading-edge if tumor cells were utilizing existing sinusoidal ECM to enter into the liver parenchyma. To this end, we analyzed the tumor leading edge in 3 representative ER-positive, chemotherapy naïve cases that displayed replacement growth as depicted in Fig. 4b, **upper panel.** We next focused specifically on Col IV, because it also a classic basement membrane protein in the normal mammary gland and is associated with resistance to anoikis in a murine liver metastasis model.^52,53^. Within the ~ 100 micron leading edge capture areas on the tumor and adjacent normal sides of the interface (Fig. 4b, lower panels), Col IV staining patterns were delineated into three categories based on the observable range of Col IV staining patterns within these 3 cases: no visible difference from normal liver (category 1), very modest thickening/diffusion (category 2), and moderate thickening/diffusion (category 3) (Fig. 4c). Within the adjacent normal liver (Fig. 4b, lower panel, region labeled “N”), we found 69% category 1, 31% category 2 and 0% category 3 staining patterns, whereas within the leading edge of the BCLM (Fig. 4b, lower panel, region labeled “T”), we found 31% category 1, 56% category 2, and 13% category 3 staining patterns (Fig. 4d). These data show that the sinusoidal Col IV staining pattern is slightly more diffuse on the tumor side compared to adjacent normal liver, implicating modest tumor cell remodeling of Col IV. In sum, these observations are consistent with tumor cell adhesion to Col IV during the establishment and/or expansion of breast cancer within the liver. A model describing a potential mechanism for BCLM replacement growth pattern is shown in (Fig. 4e).

## Discussion

To improve outcomes for women with breast cancer metastasis to the liver there is a need for improved prognostics to help identify at risk patients and effective therapeutic targets. Progress has been hindered by gaps in knowledge regarding the clinicopathologic features of primary breast cancers with a propensity to metastasize to the liver, as well as a lack of histopathologic data regarding the BCLM lesions themselves. Here, in a single, tertiary care institution study, we find our BCLM cohort is comprised mainly of patients diagnosed with early-stage, low grade, and luminal A breast cancers. These data are not in alignment with known prognostic attributes for breast cancer metastasis overall, such as ER-negative, HER2-positive, high grade and late-stage tumors. Instead, our data suggests that these classical poor prognostic features may not adequately identify breast cancer cases that progress to liver metastasis. We also found that the majority of BCLM lesions are also ER-positive, which indicates that loss of ER expression is not a requisite for BCLM. In addition, the vast majority of BCLM lesions, regardless of ER subtype, displayed a replacement growth pattern consistent with breast cancer cells using existing sinusoidal vasculature to access the liver parenchyma and establish outgrowth. Finally, patients with BCLM in our cohort were more likely to be younger, parous, and have had a recent childbirth than expected based on breast cancer demographics overall. These observations suggest that in young women, the normal liver may be a highly permissive tissue environment for breast cancer outgrowth, with liver tropism being an attribute of the liver, rather than dominant, gain-of-function attributes within the tumor cells themselves.

Our finding that patients with breast cancer liver metastases were most frequently diagnosed with luminal A cancers of low grade and early stage do not align with several published studies identifying ER-negative and HER-2 positive subtypes, higher tumor grade, and late stage at time of primary breast cancer diagnosis as predictive of metastasis in general^38,39,54^. However, only a few research groups have evaluated liver specific metastasis to date, and our study is aligned with their prior observations.

Specifically, increased prevalence of liver metastasis have been reported in younger patients^40^, young patients with early-stage ER-positive disease^16^, and patients with recent childbirth^17,20,21^. Thus, young patient age and/or proximity to recent childbirth may prove to be independent risk factors for breast cancer metastasis to the liver, with more research required to address potential causality.

Our study also adds to the understanding of the complex roles of estrogen receptors in breast cancer metastasis. Previous studies demonstrated ER heterogeneity in a primary tumor can give rise to ER-negative subsets that have increased capacity for metastasis^43,55–57^ and resistance to endocrine-therapy^58^. Here, we found 90% of ER-positive primary cases retained ER expression at the liver metastatic site. One interpretation is that these ER-positive BCLM lesions might be responsive to endocrine therapy. However, ER staining alone is insufficient to delineate between wild type, constitutively activated^59^, or mutated, non-functional ER^60^. Our observation of PR loss in ~ 50% of the ER-positive BCLM lesions is consistent with loss of ER function, as PR expression is dependent on ER signaling^61^. Of potential relevance, ER-positive postpartum breast cancers are reported to have a transcriptional profile more similiary to ER-negative breast cancer^62^, data consistent with suppression of ER signaling in the postpartum setting. A limitation of our current study is that we did not profile gene expression patterns in the BCLM lesions and thus cannot confirm reduced ER signaling in the ER + PR-lesions compared to the ER + PR+ lesions, nor could we assess potential interactions with recent childbirth. Thus, the question of whether the ER + PR- BCLM lesions are responsive to endocrine blockade therapy requires further investigation.

Our data, showing that most of the BCLM lesions displayed replacement growth with maintenance of sinusoidal architecture, may shed insight into the high incidence of metastasis arising from early stage, ER-positive breast cancers that we observed in this cohort. Replacement growth is consistent with tumor cell co-option of normal liver sinusoids for blood supply rather than gain of angiogenic capacity. Co-option of liver sinusoids during metastasis was first defined for colorectal cancer^29,49^ and is considered a “passive” mode of metastasis because it does not appear to require tumor cell-stroma crosstalk known to drive tumor angiogenesis, desmoplasia, and/or gross remodeling of organ vasculature^63–65^. The liver may be inherently optimized for “passive” tumor cell invasion because it has unique vasculature, including incomplete subendothelial basement membranes and fenestrated sinusoidal endothelium^66^. These features are anticipated to facilitate tumor cell extravasation into the liver parenchyma and permit tumor cell access to nutrients directly from the intestine, respectively^50^. In total, the emerging data on replacement growth is consistent with breast cancer metastasis to the liver being less dependent on tumor cell intrinsic biology than previously appreciated. Pre-clinical studies may support this hypothesis, as breast cancer cell lines genetically selected to have increased propensity for organ specific metastasis in murine models have been readily selected for lung^67^, bone^67^ and brain^68^, but cell lines with increased propensity for liver metastasis have been more elusive.

With the goal of identifying targetable BCLM vulnerabilities, we interrogated invasion at the breast cancer-adjacent normal liver interface. We found that the space of Disse, the space that separates sinusoidal endothelium from hepatocyte cords, contains Col IV and FN, as previously reported^69^. We extended these observations by showing that Col IV and FN are closely aligned only with the basal side of the sinusoidal endothelium, a location consistent with a role in tumor cell establishment after extravasation from the blood vessel. It is interesting to speculate that upon extravasation, tumor cell contact with Col IV provides a distinct survival advantage for low-grade luminal tumors. Col IV is an abundant basement membrane protein in normal breast that confers tissue specificity and homeostasis^52,70^, and breast cancer cells can recapitulate mammary duct morphology when plated in Col IV^71^. Thus, luminal A tumor cell interactions with sinusoidal Col IV might help explain the high numbers of luminal A BCLM cases seen in our cohort. It is noteworthy that we did observe modest Col IV remodeling within the BCLM lesions, raising the possibility that partial proteolysis of Col IV, or sinusoidal ECM more generally, may contribute to the successful establishment of BCLM. In support of this idea, denatured Col IV has been reported to have pro-tumor immunosuppressive activity in a mouse model of melanoma^72^. Further, previous work in a murine lung cancer model found that liver metastasis was dependent upon a Col IV/integrin alpha 2/FAK signaling axis, specifically through tumor cell resistance to anoikis-mediated cell death^53^. If Col IV similarly provides an immune suppressive and or survival advantage to breast cancer cells within the liver, signaling downstream of Col IV may prove to be therapeutically targetable, as previously suggested^73^. Whether these putative mechanisms for Col IV are at play in BCLM remain important areas for further investigation.

Our data also support the hypothesis that the liver metastatic niche is enhanced in young women with recent childbirth. Previous studies have reported that good prognostic criteria (stage I-II and ER-positive) breast cancers diagnosed postpartum had increased liver metastasis^17,21,74^. Further, relative increased risk for liver metastasis is highest in young patients diagnosed with early-stage disease^17,21,74,75^. With incidence trends for YOBC showing increases in stage I ER-positive diagnoses across almost all racial and ethnic groups^8^, renewed focus on the potential lethality of early-stage ER-positive YOBC and its propensity for liver metastasis are warranted. Previously work in rodent models identified weaning-induced liver involution, a tissue remodeling process that induces numerous changes to the normal liver microenvironment that are anticipated to create a pre-metastatic niche, and support liver organotropism. These changes include programmed hepatocyte cell death with potential for reduced tumor cell competition within the niche^76^, increased liver ECM deposition including Col IV^77^, reduced T cell activation^78^, increased myeloid cells with regulatory/suppressive phenotypes^79^, and broad transcriptional changes consistent with premetastatic liver niches described for gastric and pancreatic cancer^79^. Consistent with liver involution being a driving force of BCLM, in animal models of postpartum breast cancer, tumor cells have a growth advantage when implanted into the portal vein during the early involution period compared to nulliparous controls^20,78,79^. Although it is challenging to study weaning-induced liver involution in humans, liver size and metabolic output measurements across a pregnancy/wean cycle in healthy women are consistent with conservation of weaning-induced liver involution across species^80^. In addition to the idea of weaning induced involution being a “hot spot” of BCLM risk, the concept of weaning-induced involution mimicry is emerging as a potential driver of poor outcomes in breast cancer patients overall^81,82^. As such, our study positions involution biology in the liver as a construct to investigate general mechanisms of breast cancer liver tropism across patient populations.

Our study has limitations necessitating further investigations. This is a single institution study with small cohort size in need of replication. Because this study only analyzed attributes of BCLM patients, we did not assess how their demographic and clinical tumor attributes compared to breast cancer patients without liver metastasis. Additionally, the cohort analyzed was defined by availability of liver metastasis tissue, which may skew the data and restrict generalization.

In conclusion, in our BCLM cohort, classic poor prognostic tumor attributes at the time of breast cancer diagnosis do not adequately predict metastasis to the liver. Clinicians should be aware of the potential for increased risk of liver metastasis among young women, especially those diagnosed with early stage, luminal A disease, and in close proximity to recent childbirth. This PPBC subset of YOBC patients may need treatment escalation and increased surveillance. Finally, to advance our collective understanding of the enhanced liver tropism observed in young onset breast cancer patients, routine delineation of breast cancer metastasis by metastatic site, patient age, and proximity to recent childbirth are warranted.

## Supplementary Material

Supplementary Files

This is a list of supplementary files associated with this preprint. Click to download.

- SuplementaryInformationNatureCommunications.docx

## Figures and Tables

**Table 1.** Patient demographic characteristics.

Variable	Total Cases, N (%)	Cases Excluding Unknowns, N (%)
**Age, y**		
≤45	18 (38.3%)	18 (39.1%)
>45 ≤60	23 (48.9%)	23 (50 %)
>60	5 (10.6%)	5 (10.9%)
Unknown	1 (2.1%)	
**Menopausal Status**		
Pre-menopausal ≤51y	27 (57.4%)	27 (58.7%)
Post-menopausal>51y	19 (40.4%)	19 (41.3%)
Unknown	1 (2.1%)	
**Parous Status**		
Parous	39 (82.9%)	39 (90.7%)
Nulliparous	4 (8.5%)	4 (9.3%)
Unknown	4 (8.5%)	
**Postpartum Breast Cancer Status**		
PPBC	13 (27.7%)	13 (34.2%)
PPBC ≤45 y	13 (72.2%)	13 (81.25%)
Non-PPBC<45 y	3 (16.7%)	3 (18.75%)
PPBC Unknown	2 (11.1%)	

**Table 2.** Patient primary tumor clinical characteristics

Variable	Total Cases, N (%)	Cases Excluding Unknowns, N (%)
**Breast Cancer Hormone Status**		
ER Positive	40 (85.1%)	40 (86.96%)
PR+	30 (75%)	30 (75%)
PR−	10 (25%)	10 (25%)
ER Negative	6 (12.8%)	6 (13.04%)
PR−	6 (100%)	6 (100%)
Unknown	1 (2.1%)	
**Breast Cancer HER2**		
HER2 Positive	7 (14.9%)	7 (15.6%)
ER+	6 (85.7%)	6 (85.7%)
ER−	1 (14.4%)	1 (14.4%)
HER2 Negative	38 (80.9%)	38 (84.4%)
ER+	33 (86.8%)	33 (86.8%)
ER−	5 (13.2%)	5 (13.2%)
Unknown	2 (4.25%)	
**Grade**		
Grade 1	5 (10.6%)	5 (13.51%)
Grade 2	16 (34%)	16 (43.24%)
Grade 3	16 (34%)	16 (43.24%)
Unknown	10 (21.3%)	
**Stage**		
Stage I	5 (10.7%)	5 (13.2%)
Stage II	14 (29.8%)	14 (36.8%)
Stage III	7 (14.9%)	7 (18.4%)
Stage IV	12 (25.5%)	12 (31.6%)
Unknown	9 (19.1%)	
**Biological Subtype**		
Luminal A	27 (57.4%)	27 (60%)
Luminal B	6 (12.8%)	6 (13.3%)
Luminal HER2	6 (12.8%)	6 (13.3%)
HER2	1 (2.1%)	1 (2.2%)
TNBC	5 (10.6%)	5 (11.1%)
Unknown	2 (4.3%)	

**Table 3.** ER and PR expression by immunohistochemistry in paired primary breast cancer and BCLM tumors.

Primary Tumor		BCLM Tumor		
Hormone Receptor (HR)	Total	HR+	HR−	Discordance
ER+	39	35	4	4/39 (10.3%)
PR+	23	10	13	13/23 (56.5%)
PR−	10	4	6	4/10 (40%)
ER−	6	0	6	6/6 (0%)
PR+	0	0	0	0/0 (0%)
PR−	6	0	6	6/6 (0%)

## Data Availability

Data was stored using OHSU’s Advanced Computing Center Research Storage, a resource funded by an NIH S10 grant (S10OD034224) and OHSU matching funds. Materials, data, code, and associated protocols will be made promptly available to readers without undue qualifications.

## Abbreviations and Acronyms

AEC: 3–Amino 9–Ethylcarbazole
BC: Breast cancer
BCLM: Breast cancer liver metastasis
CD105: Endoglin (an accessory receptor for TGFB)
Col IV: Collagen IV
ECM: Extracellular matrix
EDTA: Ethylenediaminetetraacetic acid
ER: Estrogen receptor
FFPE: Formalin–fixed paraffin–embedded
FN: Fibronectin
GATA3: GATA binding protein 3
Hep Par 1: Hepatocyte paraffin 1
HER2: Human epidermal growth factor receptor 2
HR: Hazard ratio
ID: Identification
IHC: Immunohistochemistry
IRB: Institutional review board
Ki67: Antigen Kiel 67, proliferation marker
mIHC: Multiplex immunohistochemistry
